# Death Caused by Hemorrhage from the Vaginal Mucous Membrane

**Published:** 1857-12

**Authors:** 


ARTICLE VI.

[TRANSLATED BY DR. BYFORD.]

### DEATH CAUSED BY HEMORRHAGE FROM THE VAGINAL MUCOUS MEMBRANE.

A young spare girl, aged 14 years, in the beginning of June, 1852, was taken with bleeding from the vagina, which was slight and regarded as a menstrual effort. Ten days later, a small amount was again discharged, and on the 27th of June a larger amount was evacuated, without, however, causing much solicitude. On the 10th of July the hemorrhage was so considerable that Dr. H. Obie was called. Excepting headache, the patient experienced no pain whatever.

The loss was so great, that every half hour it was necessary to change the bedding, and the girl exhibited signs of great anæmia. All the remedies usually recommended, acids, cold applications, vaginal tampan, compression of the abdominal aorta, and (as might have been expected—TRANS.) mesmerism itself failed, and death occurred on the 16th.

Upon post-mortem examination being made, the abdominal and pelvic organs, with the exception of general bloodlessness, were quite sound. The cavity of the uterus was free from the appearance of blood and its orifice closed. But the vaginal walls were very much relaxed, and the mucous membrane separated from the subjacent tissue by ecchemosis in a great many places. No signs of ruptured vessels or loss of tissue were to be found. The abdominal aorta was empty.—*Schmidt's Jahrbucher.*

